# Cesium Lead Bromide Nanocrystals: Synthesis, Modification, and Application to O_2_ Sensing

**DOI:** 10.3390/s22228853

**Published:** 2022-11-16

**Authors:** Zhi-Hao Huang, Madhuja Layek, Chia-Feng Li, Kun-Mu Lee, Yu-Ching Huang

**Affiliations:** 1 Department of Chemical and Materials Engineering, Chang Gung University, Taoyuan City 33302, Taiwan; 2 Department of Materials Engineering, Ming Chi University of Technology, New Taipei City 24301, Taiwan; 3 School of Engineering, Brown University, Providence, RI 02912, USA; 4 Department of Pediatrics, Division of Neonatology, Chang Gung Memorial Hospital, Taoyuan City 33305, Taiwan

**Keywords:** CsPbBr_3_ nanocrystals, nanocrystal shapes, aggregation sizes, substrates, oxygen, optical gas sensor

## Abstract

The fluorescence intensity of inorganic CsPbBr_3_ (CPB) perovskite nanocrystals (NCs) decreases in the presence of O_2_. In this study, we synthesized CPB NCs with various shapes and sizes for use as optical gas sensing materials. We fabricated O_2_ gas sensors from the various CPB NCs on several porous and nonporous substrates and examined the effects of the NC shapes and aggregate sizes and the substrate pore size on the device response. Our sensor fabricated from CPB nanocrystals on a porous substrate exhibited the highest response; the porous substrate allowed the rapid diffusion of O_2_ such that the NC surface was exposed effectively to the gas. Thus, the interfacial interaction between NC surfaces and substrates is a critical factor for consideration when preparing gas sensors with a high response.

## 1. Introduction

As functional devices for detecting toxic and combustible gases in the surrounding environment, gas sensors have become an indispensable part of modern life [1,2,3]. Many sensing methods have been proposed based on semiconductor, electrochemical, and optical sensors [4,5,6]. Among them, optical gas sensing through photoluminescence (PL) quenching can provide good sensitivity, high selectivity, fast sensitivity, and a long operating lifetimes [7,8,9,10]. Nevertheless, optical gas sensors remain expensive and difficult to miniaturize, and there are display issues relating to poor thermal stability [9,11]. Oxygen (O_2_) is consumed by almost all living organisms to breath or produce energy. Under extreme conditions, excessive levels of O_2_ can seriously affect the central nervous system, eyes, and lungs, causing cell damage or death (so-called O_2_ poisoning) [12]. Therefore, monitoring O_2_ is important not only to ensure appropriate cellular respiration but also in industrial processes. Several O_2_-sensitive molecules have been used in O_2_ monitoring devices, including metal oxide semiconductors [13,14], fullerenes [15,16], and metal–organic frameworks (MOFs). Recently, luminescent nanocrystals (NCs) have become emerging alternative O_2_-sensitive materials because of their excellent optical properties and high surface-to-volume ratios [17,18,19].

Lead halide perovskite materials have been used as photoactive layers in various photonic and optoelectronic devices, including lasers [20], solar cells [21,22], light emitting diodes (LEDs) [23,24], and photodetectors [25,26]. Perovskite materials possess the chemical structure ABX_3_, where A, B, and X are typically organic or inorganic cations, metal cations, and halide anions, respectively. Organic/inorganic hybrid halide perovskites can be used as gas sensors for the detection of NH_3_ [27], HCl [28], and O_2_ [29], due to their high charge mobilities, tunable spectra, high-intensity PL emissions, and good defect tolerance [30,31]. Recently, researchers have been paying greater attention to the technological development of all-inorganic perovskites. Because they lack organic molecules in their chemical structures, all-inorganic perovskites possess structural and environmental stability superior to that of organic/inorganic hybrid halide perovskites. For example, cesium lead bromide (CsPbBr_3_, CPB) has been studied broadly because of its high charge mobility, stability, and photoresponsivity [32,33,34]. Recently, Chen et al. used CPB NCs to detect toxic and corrosive HCl vapors at concentrations as low as 5 ppm through visual observation of a facile gaseous anion-exchange reaction [28]. Wu et al. prepared an impedance-type humidity sensor based on CPB NCs that displayed excellent repeatability, low hysteresis, and good stability in terms of rapid response and recovery at room temperature [35]. Kim et al. found that p-type CPB NCs possessed high gas-sensing selectivity for the detection of NO_2_; sequential treatment of CPB NCs thin films with methyl acetate soft soaking and Ostwald ripening improved their transport properties and dramatically enhanced the carrier lifetime from 11.56 to 112.5 ns [36]. Although some reports have appeared describing the use of CPB NCs in gas sensors, there have been no deep explorations of their applications. In addition, O_2_ sensors have continued to exhibit issues that have limited their commercial application, including a lack of selectivity, response to humidity, slow recovery times, short lifetimes, low stability, and high cost.

In this study, we used ligand-assisted reprecipitation (LARP) to synthesize CPB NCs having various aggregate sizes and shapes, and then investigated the effects of these structures on their O_2_ sensing ability. The response of resulting devices depended greatly on the thickness of the deposited film and the pore size. Therefore, we deposited the various CPB NCs on nonporous and porous substrates to find matching substrates and NCs that enhanced the response of O_2_ detection. Through this simple and inexpensive method for the synthesis and modification of CPB NCs, we developed a facile sensor device that appears to be conducive to the construction of cost-effective gas sensors in the future.

## 2. Experimental Section

### 2.1. Materials

Cesium bromide (CsBr, 99.9%), lead (II) bromide (PbBr_2_, 99.9%), oleic acid (C_18_H_34_O_2_, 65–80%), and *n*-hexane (C_6_H_14_, anhydrous, 95%) were purchased from Sigma–Aldrich. Oleylamine (C_18_H_35_NH_2_, 80–90%) and acetone (CH_3_COCH_3_, 99.8%, extra dry) were obtained from Acros Organics. Octylamine (C_8_H_19_N, 99%) and toluene (C_7_H_8_) was purchased from Alfa Aesar and Tedia, respectively. Filter paper (pore size: 5–6 µm) and membrane filters (pore size: 2 µm–200 nm) were acquired from Advantec MFS. An anodic aluminum oxide (AAO) membrane filter (pore size: 200 nm) was purchased from Whatman. All materials and substrates were used as received without any further purification.

### 2.2. Synthesis and Shape Modification

The synthesis of the CPB NCs was performed mainly as described previously [37], but with slight modifications to obtain CPB NCs with different shapes. Three solutions were prepared: 0.3 mmol CsBr in deionized water (500 µL) (solution 1); 0.3 mmol PbBr_2_ in dimethylformamide (DMF, 500 µL) (solution 2); and *n*-hexane (10 mL) mixed with oleic acid (2 mL) and octylamine (0.25 mL) (solution 3). Solutions 1 and 2 were added to solution 3 (dropwise) followed by rapid addition of acetone (8 mL). The mixture was then centrifuged (6000 rpm, 10 min). The precipitate was re-dissolved in *n*-hexane (2 mL) and centrifuged again (6000 rpm, 10 min). The supernatant was collected. This procedure was repeated two more times to produce two more colloidal solutions. Post-synthesis modification was performed based on the procedure described by Fanizza, Elisabetta, et al. [38]. Oleic acid (25 and 50 µL; providing samples Olac 25 and Olac 50, respectively) and oleylamine (25 and 50 µL; providing samples Olam 25 and Olam 50, respectively) were separately added dropwise into the as-synthesized CPB NC solutions (1 mL). The resulting solutions were centrifuged (1000 rpm, 10 min) at room temperature (25 °C) and the colloidal solids were collected.

### 2.3. Device Fabrication and Characterization

**Figure 1:**
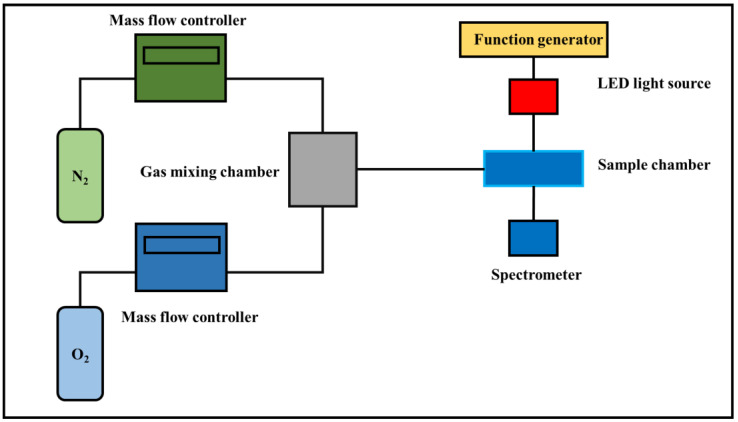
Schematic for optical gas sensing.

Solutions of the CPB NCs, Olac 25, Olac 50, Olam 25, and Olam 50 were spin-coated onto glass, filter paper, a membrane filter, and an AAO membrane filter at spin-coating rates of 1000, 2000, 3000, 4000, and 5000 rpm. The resulting samples were dried at room temperature to obtain the sensing layers. UV–Vis spectrometry (JASCO, V-770) was used to measure the film absorption behavior. Transmission electron microscopy (TEM, JEOL, JEM-2100) was used to identify the shapes of the CPB NCs. The particle size distributions were measured using a particle size analyzer (Malvern Panalytical, Zetasizer 2000) and scanning electron microscopy (SEM, Phenom Pro-X). Figure 1 provides a schematic representation of the gas sensing process. The O_2_ and N_2_ gas flows were controlled using a mass flow controller (Aalborg Instruments and Controls, Model GFC 17). The gases were mixed in the chamber and the flow rates were changed as required. Fluorescence excitation was provided by an LED with a central wavelength of 405 nm and output power of 10 mW/cm^2^ driven by a waveform generator (TGA1240, Thurlby Thandar Instruments); the spectrum was recorded using a USB4000 fiber optics spectrometer (U.S Ocean Optics). All measurements performed done at 25 °C.

### 2.4. Mechanism of Sensing

Perovskite materials have been extensively used for oxygen sensing. Stoeckel et al., in 2017, used CH_3_NH_3_PbI_3_ for oxygen gas detection using the change of output current [29]. As the oxygen concentration decreased, the device output current also decreased. Once the O_2_ was withdrawn from the sensing environment, the resistance of the film went back to its original value, indicating the reversibility of the sensor. The sensor showed a fast response (~0.4 s) and had a detection limit of ~70 ppm. The authors concluded that the oxygen diffuses inside the crystals and heals the iodine vacancies on the perovskite surface. These vacancies act as charge trap state. In the presence of oxygen, the trap heals and the availability of the charges increases. This in turn increases the output current.

## 3. Results and Discussion

### 3.1. Effect of Different NC Shapes on Device Response

**Figure 2:**
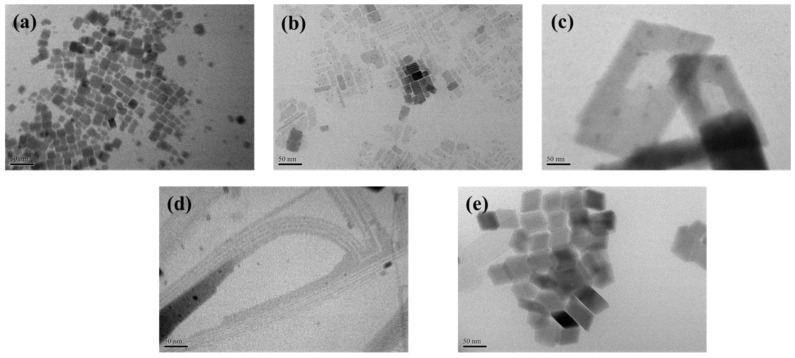
Transmission electron microscopy images of (**a**) as-synthesized NCs, (**b**) Olac 25, (**c**) Olac 50, (**d**) Olam 25, and (**e**) Olam 50.

The TEM images (Figure 2a) and diffraction pattern (Figure S1) of the CPB NCs revealed a cube-shaped polycrystalline structure that had a (100) interplanar spacing of 0.42 nm. We synthesized CPB NCs with various shapes to investigate the effect of the shape on the device response. Upon adding various amounts of oleic acid and oleylamine, the originally nanocube-structured CPB NCs transformed into a diverse array of nanostructures. Figure 2b,c present TEM images of the Olac 25 and Olac 50 NCs, respectively; their shapes changed from nanocubes into nanosheets of various sizes. The Olam 25 and Olam 50 NCs possessed nanowire (Figure 2d) and rhombus (Figure 2e) structures, respectively. Figure S2 displays the colors and fluorescence properties of solutions of the variously shaped NCs. The Olac 25 and Olac 50 solutions were yellow, while the Olam 25 and Olam 50 solutions were bright green. Notably, after modification for 3 h, the color of the Olam 50 solution changed from bright green to white, with the disappearance of its fluorescence. Thus, because of instability, we did not use Olam 50 to fabricate a gas sensor. Figure S3 presents the size distributions of the synthesized and modified NCs; the average particle sizes of the as-synthesized, Olac-25, Olac-50, and Olam-25 NCs were 47.32 ± 0.08, 49.72 ± 0.26, 74.3 ± 0.06, and 32.1 ± 0.16 nm, respectively.

**Figure 3:**
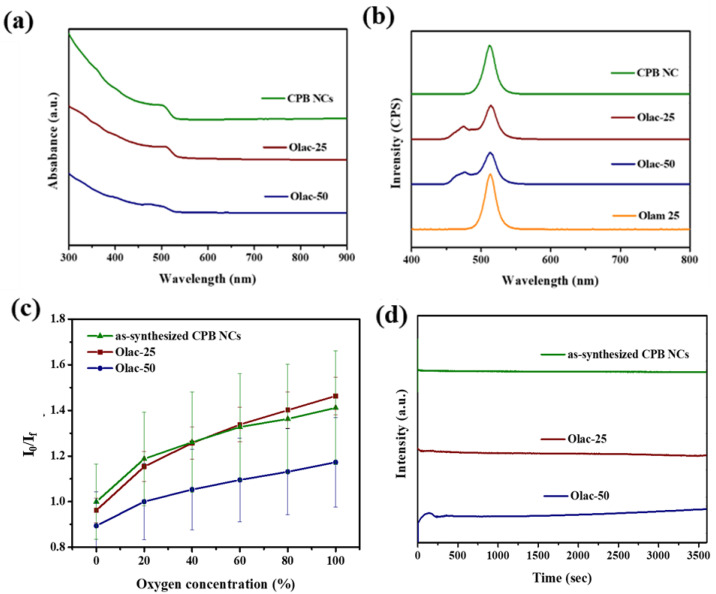
(**a**) UV–Vis absorbance, (**b**) emission spectra of as-synthesized CPB NCs (olive line), Olac 25 (wine line), Olac 50 (navy line), and Olam 25 (orange line) samples. (**c**) Response and (**d**) photostability of as-synthesized CPB NCs (olive line), Olac 25 (wine line), and Olac 50 (navy line) samples.

Figure 3a,b displays the UV–Vis absorption and PL spectra of the CPB NCs. The CPB NCs featured a typical absorption peak near 500 nm, with slight changes in the absorption spectra of the Olac 25 and Olac 50 samples. The PL emission wavelength of the as-synthesized CPB NCs was 512 nm; for Olac 25, Olac 50, and Olam 25 it had slightly shifted to 514 nm. In addition, the spectra of Olac 25 and Olac 50 also featured a PL emission signal at 475 nm, due to the presence of nanosheets, which might explain the absence of any peaks below 500 nm for Olam 25. We spin-coated the various solutions onto a glass substrate to fabricate gas sensor devices, which we placed in a chamber and exposed to atmospheres of various N_2_/O_2_ mixtures and recorded the changes in fluorescence. Figure S4 displays the changes in fluorescence intensity of these sensors with respect to the O_2_ concentration. The fluorescence intensity decreased upon increasing the O_2_ concentration. Response is a parameter used to assess the ability of a gas sensor to detect a particular analyte. The physical and chemical properties of a sensor change in the presence of a gas [39]. In this work, we defined the response as the ratio of the initial and final values of the measured parameters of the gas sensor. The formula used to determine the response is:

R = *I*_0_*/I_f_* (1)

where R is the response, *I*_0_ is the initial intensity in absence of analyte, and *I_f_* is the intensity in the presence of a particular analyte. The measured fluorescence intensities of the devices in the chamber containing 100% N_2_ and 100% O_2_ are reported herein as the initial (*I*_0_) and final (*I_f_*) fluorescence intensities, respectively. The response of each device was identified by the ratio of *I*_0_ and *I_f_*. Because of collisional interactions between O_2_ gas and the CPB NCs, the molecules of O_2_ removed photogenerated electrons from the conduction band, resulting in a decrease in fluorescence intensity for the CPB NCs in the presence of O_2_ [24]. Figure 3c reveals the response of the as-synthesized Olac 25 and Olac 50 CPB NC samples. The fluorescence peak of the Olam 25 sample was indistinguishable from the error signal, due to its low emission (Figure S4d). The response of the as-synthesized CPB nanocubes was higher than that of the Olac-modified samples. The maximum response achieved was 1.6. We tested the photostability of the sensors by subjecting them to continuous UV excitation and recording the changes in fluorescence intensity. After 1 h, the changes in fluorescence intensity of the CPB nanocubes and Olac 25, and Olac 50 samples, as shown in Figure 3d, were 1.10, 1.03, and 0.92%, respectively. Thus, these gas sensors were stable towards continuous UV excitation.

### 3.2. Effect of Aggregation Size of CPB NCs on Device Response

**Figure 4:**
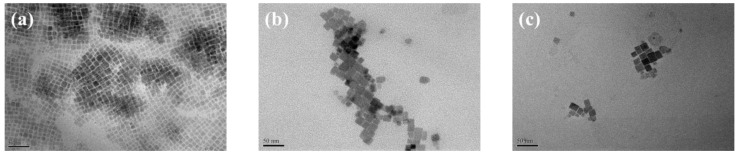
TEM images of (**a**) Agg-1, (**b**) Agg-2, and (**c**) Agg-3 samples.

**Figure 5:**
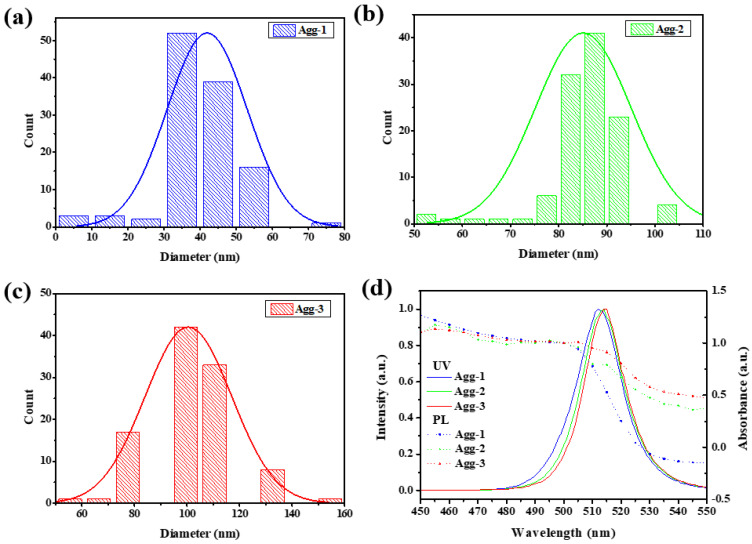
Particle size distributions of (**a**) Agg-1 (**b**) Agg-2, and (**c**) Agg-3 samples. (**d**) UV–Vis absorbance and emission spectra of Agg-1 (blue line), Agg-2 (green line), and Agg-3 (red line) samples.

We applied CPB NCs with three different aggregate (Agg) sizes to study its effect on device response. The particle size of the CPB NCs, determined from TEM images (Figure 4), was approximately 15 nm for all of these aggregates. Figure 5a–c present the particle size distributions of the three different NC solutions. The aggregate sizes in Agg-1, Agg-2, and Agg-3 were approximately 44, 88, and 101 nm, respectively. Figure 5d displays the UV–Vis absorption and PL spectra of these NCs. A shoulder appeared near 500 nm in the spectra of all of the NC Agg solutions, but the band gaps were not significantly different, indicating that the structures of the CPB NCs did not change upon varying their aggregate sizes. The emission peaks of Agg-1, Agg-2, and Agg-3 appeared at 512, 514, and 515 nm, respectively, with full widths at half maximum (FWHMs) of 19.46, 19.12, and 19.39 nm, respectively. A slight red-shift of the emission peak occurred upon increasing the aggregate size. Figure S5 reveals the sensitivities of the gas sensor fabricated from Agg-1 achieved a maximum response of 1.6; in contrast, the sensors prepared from Agg-2 and Agg-3 displayed an extremely low response, mainly because their emission intensities were too low to effectively distinguish the emission peak of the sample from error signals. This behavior implies that a large aggregate size led to a significant decrease in the interfacial contact area, thereby inhibiting interactions between the gas and the NCs and dramatically degreasing the response of the sensors.

### 3.3. Effect of Substrate on Device Response

**Figure 6:**
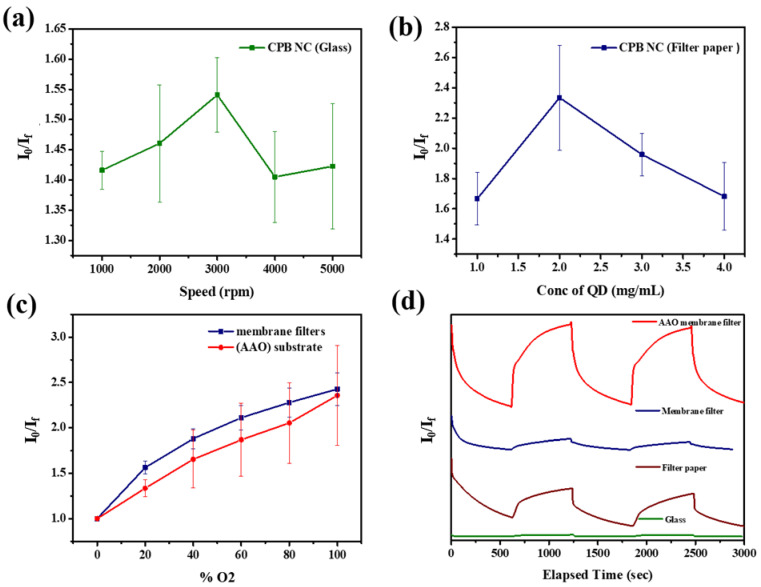
The different spin speed on the device sensitivity. The sensitivities of the sensors fabricated from (**a**) glass and (**b**) filter paper varies with the concentration of CsPbBr_3_ solutions. (**c**) The sensitivity of the membrane filters with pore sizes of ~2 µm and 200 nm (Anodic aluminum oxide (AAO) substrate) as the sensor substrates. (**d**) The response and recovery behaviors of all the devices.

Because the interfacial area of the CPB NCs greatly influenced the response, we employed substrates with various surficial morphologies to modify the interfacial areas of the CPB NCs with the goal of enhancing the device response. Accordingly, we deposited the CPB solution onto glass, filter paper, a membrane filter, and an AAO membrane filter to fabricate a range of gas sensor devices. We placed these devices in a chamber, exposed them to various N_2_/O_2_ mixing atmospheres, and recorded the changes in fluorescence intensity. For the gas sensor fabricated on the glass substrate, we spin-coated the films at various spin rates to evaluate the effect of film thickness on the device response (Figure 6a). Interestingly, the film thickness had little effect on the response. In general, a thicker film should result in higher response, due to greater fluorescence quenching. Our observation of only slight changes in response with respect to film thickness implies that CPB aggregation occurred in these films. With greater aggregation in thicker films, O_2_ molecules would have difficulty interacting with the sensing materials, leading to a lower response.

Previous reports have revealed that porous substrates can improve the response of sensors as a result of increases in the permeability of the gas and the reaction area [40]. In an attempt to further improve the response of the CPB sensors, we used filter paper with a pore size of 5–6 µm as a substrate. Unlike the glass substrate, we could not spin-coat CPB films onto the filter paper; therefore, we formed CPB films by drop-coating solutions of CPB at various concentrations onto the filter paper. Figure 6b reveals that the response of the sensors fabricated using filter paper varied with respect to the concentration of the CPB solution. Upon increasing the concentration from 1 to 2 mg/mL, the average response increased from 1.67 to 2.33. Nevertheless, further increases in the solution concentration to 3 and 5 mg/mL caused the average response of the sensor to decrease to 1.95 and 1.65, respectively. The sensitivities of the CPB sensor deposited on the filter paper were much higher than those of the sensor on the glass substrate, consistent with the greater permeability. The strong decease in the emission intensity in the presence of O_2_ gas indicated that the molecules of O_2_ directly extracted photogenerated electrons from the conduction band. The high response of the CPB sensors fabricated using filter paper resulted from the increases in the amount of sensing material and the reaction area.

To further investigate the effect of the pore size of the substrate on the response, we used membrane filters with pore sizes of approximately 2 µm and 200 nm (AAO substrate) as sensor substrates. Figure 6c presents the change in response with respect to the CPB NCs concentration; the trend was the same as that for the devices fabricated using filter paper. When the NC concentration was 2 mg/mL, the highest average response reached 2.7; this value was higher than that of the sensors prepared using filter paper. In contrast, the average response of the sensor fabricated on the AAO substrate with a pore size of 200 nm was approximately 2.9 when the NC concentration was 2 mg/mL. The result is consistent with the notion that porous substrates can improve device response. In addition to response, sensor devices should display good response and recovery behavior. Initially, we placed our devices in a 100% N_2_ atmosphere for 10 min, and then changed the atmosphere to 100% O_2_. Ideally, the fluorescence intensities of the devices would decrease in the presence of O_2_ and return to their original values upon changing the atmosphere to 100% N_2_. Figure 6d presents the response and recovery behavior of all of our devices; Table S1 lists the response and recovery times. The gas sensor fabricated on the glass substrate operated with short response and recovery times, indicating that the gas could react faster with the sensing material because of the flat surface. Because the gas sensors fabricated using the porous substrates contained more sensing materials and featured longer gas diffusion paths, their operation was characterized by longer response and recovery times. Figure S6 reveals that the relative fluorescence intensity of the CPB NCs decreased by 1.1% after 1 h of continuous UV excitation. Thus, this sensor can be considered to have reasonable photostability during its operation. Table S1 summarizes the performance of state-of-the-art oxygen sensor.

**Figure 7:**
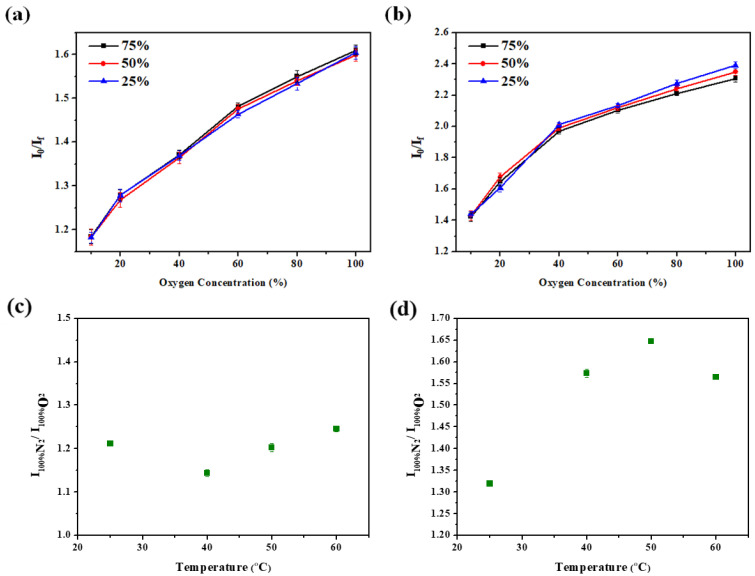
Responses of the gas sensor at different relative humidity and temperatures. Gas sensors are deposited on the glass (**a**,**c**) and filter paper (**b**,**d**) substrates.

Finally, Figure 7 reveals the effect of humidity and temperature on the response of the gas sensors deposited on the glass and filer paper substrates. Figure 7a,b show that these gas sensors exhibit similar responses under different relative humidity atmospheres. This result indicates that our gas sensor has a fairly stable response even in a high humidity environment. Figure 7c illustrates that the response of the glass-based gas sensor does not show an obvious change when the operating temperature is increased from 25 °C to 60 °C. However, the response of the sensors fabricated on the filter paper substrate gradually increased with rising temperature, with the highest response at 50 °C (Figure 7d). The increasing response implies that the porosity of the substrate is affected by the operating temperature.

## 4. Conclusions

We have synthesized various CPB NCs for use as O_2_ gas sensors and investigated the effect of the NC shape and aggregate size on the device response. We found that cubic-shaped NCs that have smaller aggregate sizes possessed a higher response. Furthermore, we examined the effects of the pore sizes of four substrates on the response of the sensors. The response of the sensors fabricated using porous substrates was higher than that of those fabricated using nonporous substrates. In addition, the response increased upon decreasing the pore size, mainly due to increases in the diffusivity of the gas and the reaction area. These key influencing factors allowed us to obtain high-efficiency CPB NC gas sensors.

## Data Availability

Not applicable.

## Supplementary Materials

The following supporting information can be downloaded at: https://www.mdpi.com/article/10.3390/s22228853/s1, Figure S1: Transmission electron microscopy images of (a) as-synthesized NCs, (b) Olac 25, (c) Olac 50, (d) Olam 25, and (e) Olam 50; Figure S2: Transmission electron microscopy diffraction patterns of (a) as-synthesized NCs, (b) Olac 25, (c) Olac 50, (d) Olam 25, and (e) Olam 50; Figure S3: Pictures of NC solutions (a,c) and solutions illuminated under UV light (b,d). Sol-1~ 5 are as-synthesized, Olac-25, Olac-50, Olam-25, and Olam-50 solutions, respectively. (a,b) are the fresh solutions, and (c,d) are the solutions after standing for 3 h; Figure S4: Particle size distributions of (a) as-synthesized NCs, (b) Olac-25, (c) Olac-50 and (d) Olam 25; Figure S5: Change in emission intensity of (a) as-synthesized CPB NCs, (b) Olac 25, (c) Olac 50, and (d) Olam 25 samples at different O_2_ concentrations; Figure S6: TEM images of (a) Agg-1, (b) Agg-2, and (c) Agg-3 samples; Figure S7: Sensitivity of Agg-1 sample at different O_2_ concentrations; Figure S8: Photostability of CPB NCs fabricated on the AAO membrane filter; Table S1: The response and recovery behaviors of all the devices, and the response and recovery time.
