# Supplementary material for: Cesium Lead Bromide Nanocrystals: Synthesis, Modification, and Application to O2 Sensing
Source: Sensors (Basel). 2022 Nov 16;22(22):8853. doi: 10.3390/s22228853 (PMC9698211; doi:10.3390/s22228853)
Supplement: Supplementary file 1 [file sensors-22-08853-s001.zip › sensors-2012972-supplementary.pdf]

Supporting Information

Cesium Lead Bromide Nanocrystals: Synthesis, Modification, and Application to O_2 Sensing

Zhi-Hao Huang [1], Madhuja Layek [2,3], Chia-Feng Li [2], Kun-Mu Lee [1,4,*] and Yu-Ching Huang [2,*]

1 Department of Chemical and Materials Engineering, Chang Gung University, Taoyuan City 33302, Taiwan;
2 Department of Materials Engineering, Ming Chi University of Technology, New Taipei City 24301, Taiwan
3 School of Engineering, Brown University, Providence, RI 02912, USA
4 Department of Pediatrics, Division of Neonatology, Chang Gung Memorial Hospital, Taoyuan City 33305, Taiwan
* Correspondence: kmlee@mail.cgu.edu.tw (K.-M.L.), huangyc@mail.mcut.edu.tw (Y.-C.H.)

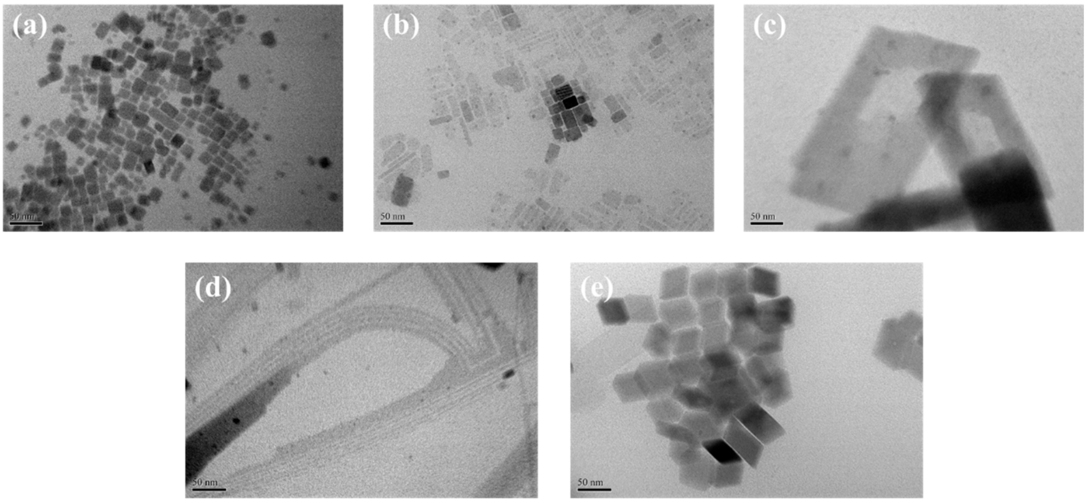

Figure S1. Transmission electron microscopy images of (a) as-synthesized NCs, (b) Olac 25, (c) Olac 50, (d) Olam 25, and (e) Olam 50.

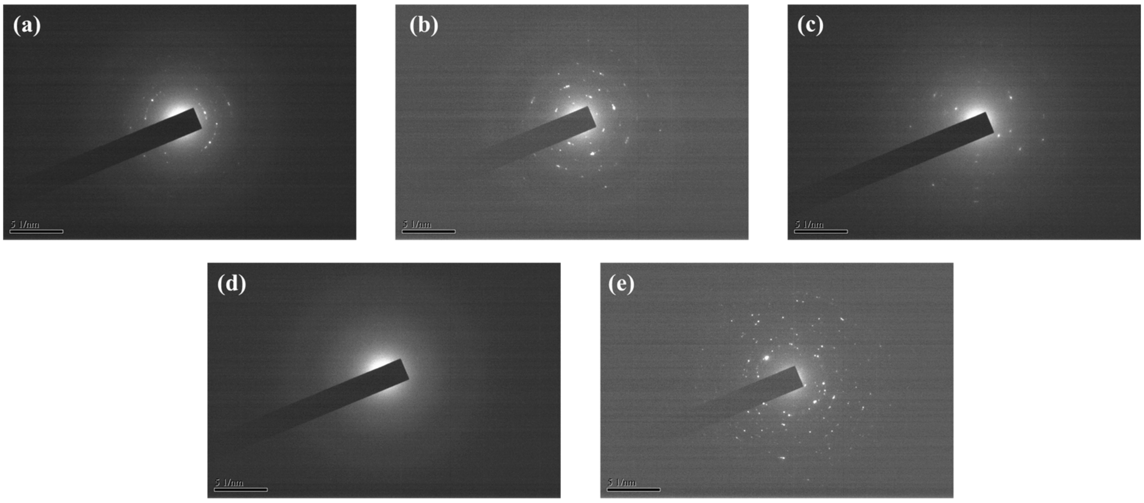

Figure S2. Transmission electron microscopy diffraction patterns of (a) as-synthesized NCs, (b) Olac 25, (c) Olac 50, (d) Olam 25, and (e) Olam 50.

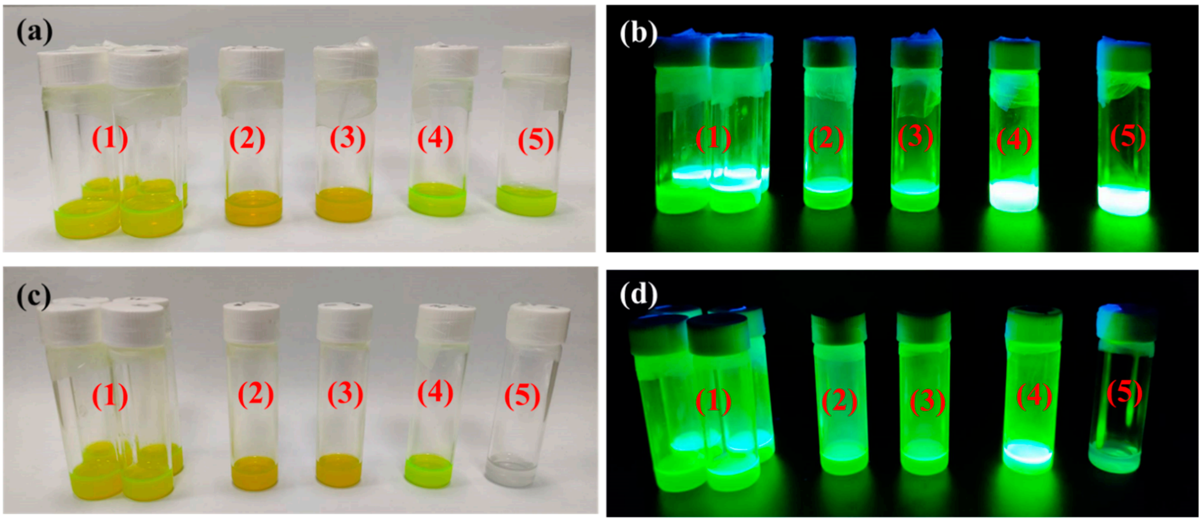

Figure S3. Pictures of NC solutions (a, c) and solutions illuminated under UV light (b, d). Sol-1~ 5 are as-synthesized, Olac-25, Olac-50, Olam-25, and Olam-50 solutions, respectively. (a,b) are the fresh solutions, and (c, d) are the solutions after standing for 3 hours.

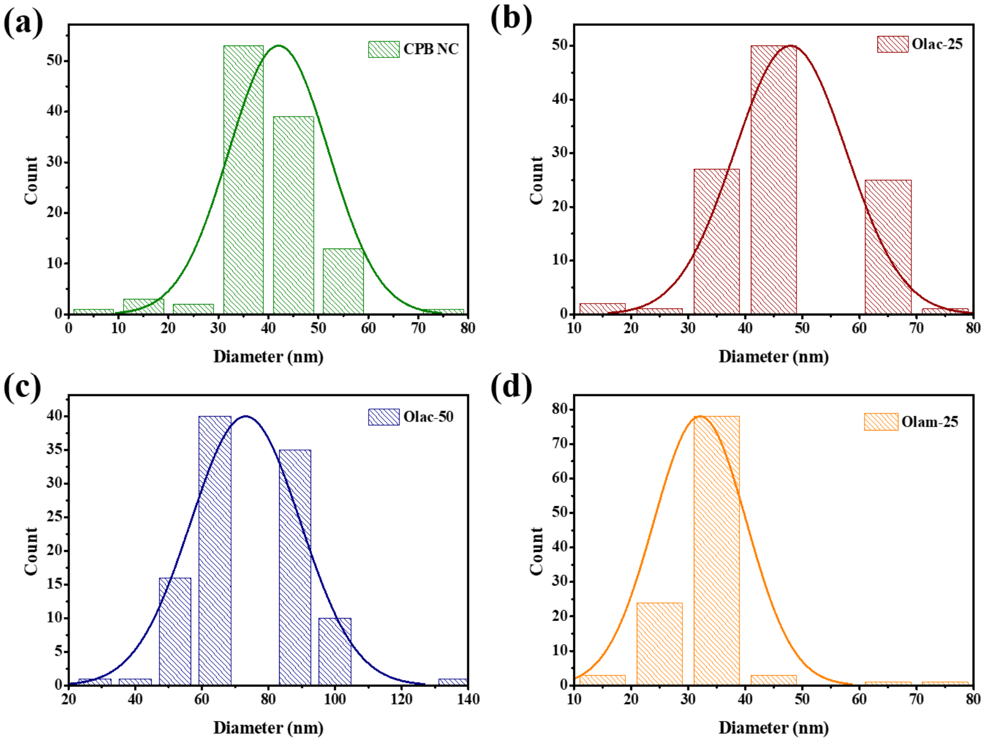

Figure S4. Particle size distributions of (a) as-synthesized NCs, (b) Olac-25, (c) Olac-50 and (d) Olam 25.

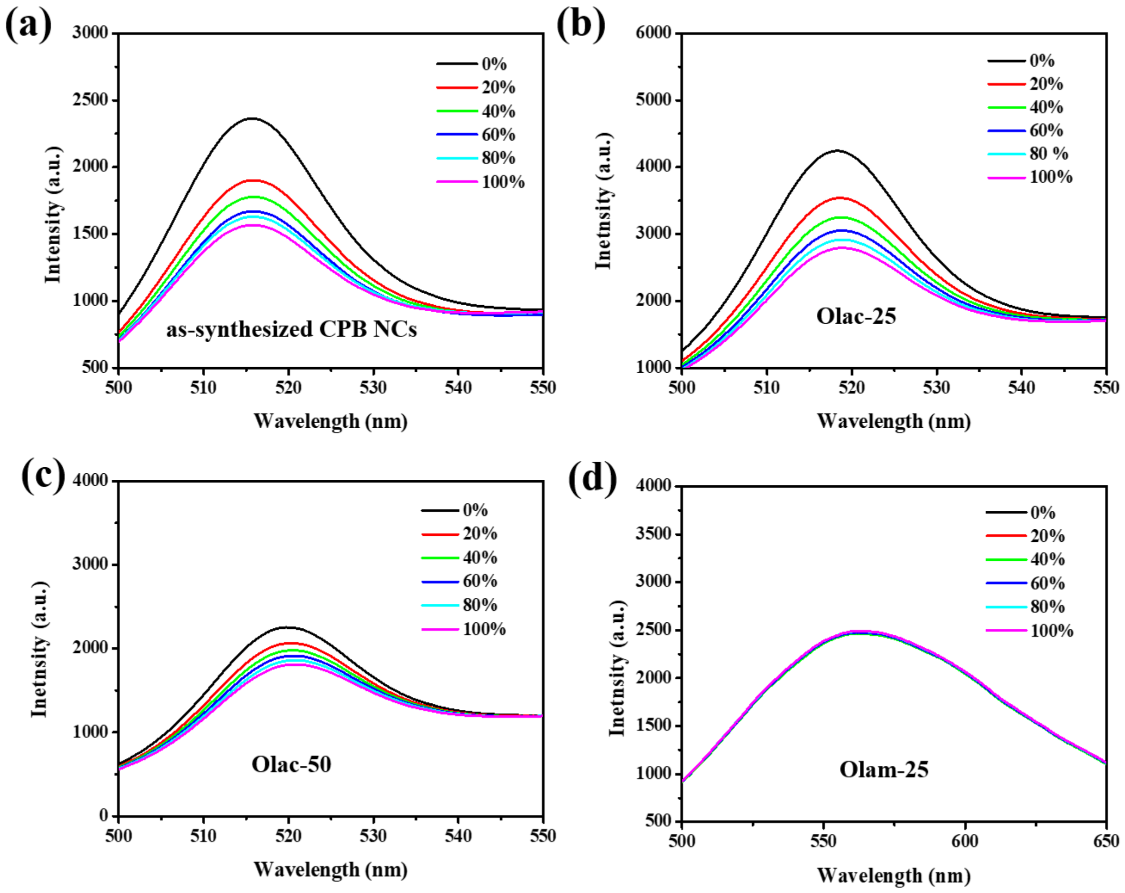

Figure S5. Change in emission intensity of (a) as-synthesized CPB NCs, (b) Olac 25, (c) Olac 50, and (d) Olam 25 samples at different O_2 concentrations.

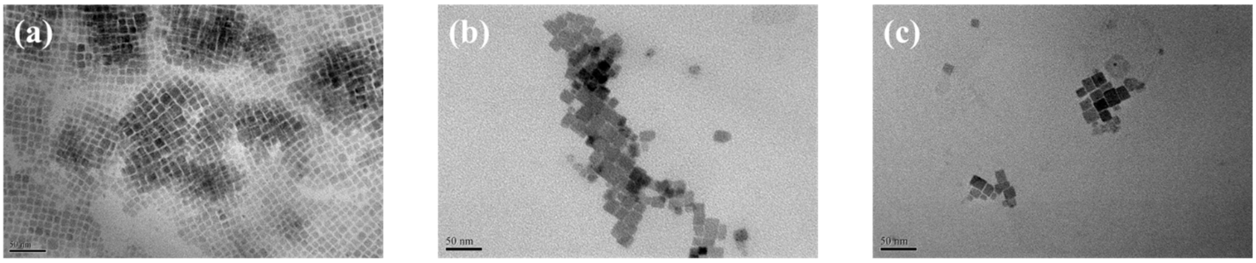

Figure S6. TEM images of (a) Agg-1, (b) Agg-2, and (c) Agg-3 samples.

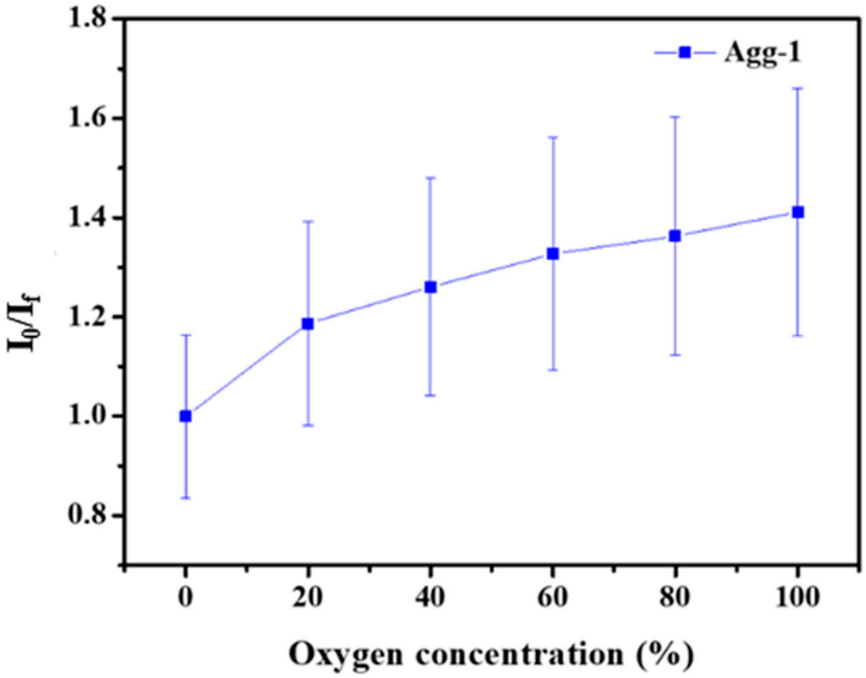

Figure S7. Sensitivity of Agg-1 sample at different O_2 concentrations.

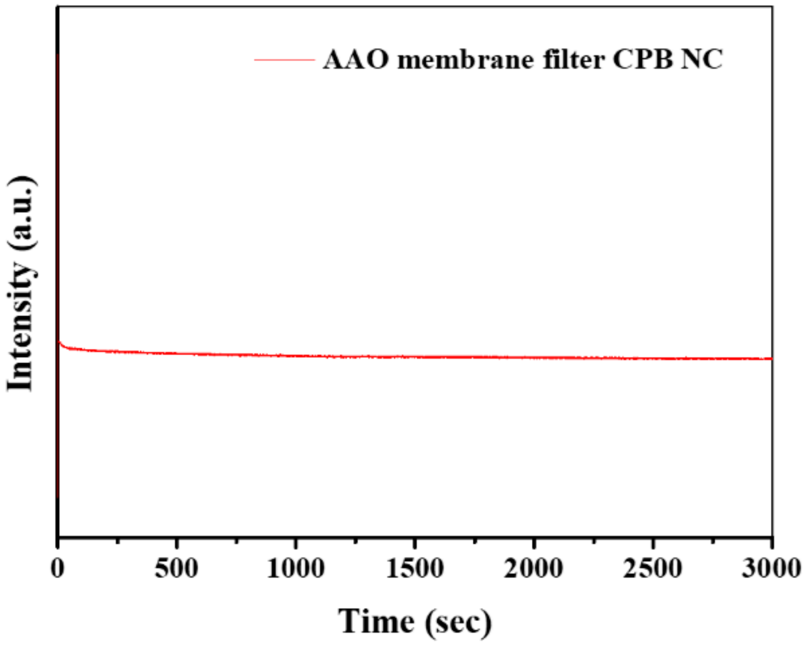

Figure S8. Photostability of CPB NCs fabricated on the AAO membrane filter.

Table S1

The response and recovery behaviors of all the devices, and the response and recovery time.

Substrate	Response Time (min)	Recovery Time (min)
Glass	2.25	3.88
Filter paper	6.58	2.58
Membrane filter	5.49	2.02
AAO membrane filter	6.55	4.57
